# An affordable detection system based on RT-LAMP and DNA-nanoprobes for avian metapneumovirus

**DOI:** 10.1007/s00253-024-13243-x

**Published:** 2024-07-10

**Authors:** Pablo Cea-Callejo, Sonia Arca-Lafuente, Esperanza Gomez-Lucia, Ana Doménech, Mar Biarnés, Angela Blanco, Laura Benítez, Ricardo Madrid

**Affiliations:** 1 BioAssays SL. Parque Científico de Madrid, Madrid, Spain; 2 Research Group of “Animal Viruses” of Complutense University of Madrid, Madrid, Spain; 3 Deparment of Animal Health, Veterinary Faculty, Complutense University of Madrid (UCM), Madrid, Spain; 4 Centro de Sanidad Avícola de Cataluña y Aragón (CESAC), Reus, Spain; 5 Department of Genetics, Physiology, and Microbiology, School of Biology, Complutense University of Madrid (UCM), Madrid, Spain

**Keywords:** Molecular detection, Reverse transcription loop mediated isothermal amplification (RT-LAMP), Nanoprobes, Avian metapneumovirus (aMPV), Point of Care (POC) test

## Abstract

**Abstract:**

Airborne animal viral pathogens can rapidly spread and become a global threat, resulting in substantial socioeconomic and health consequences. To prevent and control potential epidemic outbreaks, accurate, fast, and affordable point-of-care (POC) tests are essential. As a proof-of-concept, we have developed a molecular system based on the loop-mediated isothermal amplification (LAMP) technique for avian metapneumovirus (aMPV) detection, an airborne communicable agent mainly infecting turkeys and chickens. For this purpose, a colorimetric system was obtained by coupling the LAMP technique with specific DNA-functionalized AuNPs (gold nanoparticles). The system was validated using 50 different samples (pharyngeal swabs and tracheal tissue) collected from aMPV-infected and non-infected chickens and turkeys. Viral detection can be achieved in about 60 min with the naked eye, with 100% specificity and 87.88% sensitivity for aMPV. In summary, this novel molecular detection system allows suitable virus testing in the field, with accuracy and limit of detection (LOD) values highly close to qRT-PCR-based diagnosis. Furthermore, this system can be easily scalable to a platform for the detection of other viruses, addressing the current gap in the availability of POC tests for viral detection in poultry farming.

**Key points:**

*•aMPV diagnosis using RT-LAMP is achieved with high sensitivity and specificity.*

*•Fifty field samples have been visualized using DNA-nanoprobe validation.*

*•The developed system is a reliable, fast, and cost-effective option for POCT.*

**Graphical Abstract:**

**Figure:**
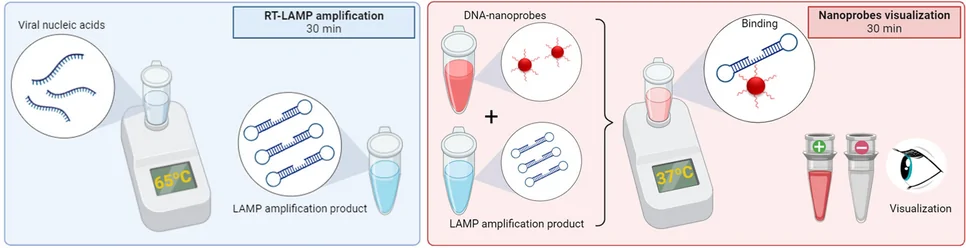

**Supplementary Information:**

The online version contains supplementary material available at 10.1007/s00253-024-13243-x.

## Introduction

Avian metapneumovirus (aMPV) infection is considered one of the most economically important upper respiratory tract diseases in poultry (Kaboudi and Lachhbeb 2021; Salles et al. 2023). Early described as the turkey rhinotracheitis (TRT) virus, it primarily affects turkeys but can also trigger the onset of swollen head syndrome (SHS) in chickens. aMPV belongs to the Pneumoviridae family, with a non-segmented, negative-stranded RNA genome containing eight genes in the following order: nucleoprotein (N), phosphoprotein (P), matrix protein (M), fusion protein (F), second matrix protein (M2), small hydrophobic protein (SH), surface glycoprotein (G), and RNA-dependent RNA polymerase (L). According to the G-gene sequence, up to six subtypes have been described so far: aMPV-A, aMPV-B, aMPV-C, aMPV-D, and two subtypes recently identified (Easton et al. 2004; Salles et al. 2023).

The diagnosis of this disease poses a challenge due to the mild or non-specific signs observed after infection. Moreover, various avian respiratory diseases manifest clinical signs closely resembling those of TRT, such as avian infectious laryngotracheitis (ILT), avian infectious bronchitis (IB), or Newcastle disease (ND), impeding its precise clinical diagnosis as a result. Viral isolation can be time-consuming and requires further research efforts. Serology offers a faster solution, but it is not always trustworthy since aMPV genetic differences can hinder its sensitivity, and it only gives retrospective information (Cook and Cavanagh. 2002). Molecular assays represent fast, useful, specific, and sensitive diagnostic techniques. To date, both conventional RT-PCR and qRT-PCR-based methods have been devised not only for its detection but also for subtype differentiation (Salles et al. 2023).

However, these PCR methods require specialized training and expensive equipment, which can limit their implementation in the field. Thus, there is a growing interest in developing alternative, robust, and reliable molecular diagnostic platforms that exploit the reverse-transcription loop-mediated isothermal amplification (RT-LAMP) strategy as a useful tool for the detection of RNA viruses in point-of-care (POC) settings. The specificity and sensitivity of RT-LAMP are potentially comparable to PCR-based diagnostics, but with a reduced reaction time and easier visualization. These features make RT-LAMP-based techniques a helpful monitoring strategy at any under-equipped veterinary hospital, but also on poultry farms. Indeed, different LAMP-based techniques have been recently developed for the diagnosis of different avian respiratory viruses such as IBV and ILTV (Wu et al. 2019; Yu et al. 2021; Padzil et al. 2021). Nevertheless, as of now, there is no RT-LAMP approach for aMPV detection as a POC test.

On the other hand, LAMP reaction readout methods like pH indicators, such as hydroxynaphthol blue or metal indicators (Thompson and Lei. 2020; Juscamayta-López et al. 2021; Nawattanapaiboon et al. 2021; Trassante et al. 2021; Raddatz et al. 2022; Goto et al. 2009), are unable to distinguish between specific and unspecific products. In fact, LAMP assays often face challenges with contaminations and false positives, attributed to their heightened sensitivity. Progress in single-step techniques and direct readouts may be pivotal in improving the method for practical on-farm applications (Padzil et al. 2021). To overcome these limitations, we propose using specific DNA-nanoprobes as gold nanoparticles (AuNPs) functionalized with specific oligonucleotides against discrete sequences of aMPV RNA. Because of their optical properties, colloidal AuNPs have recently gained popularity as a promising POC test based on colorimetric assays in combination with portable devices for the detection of pathogens (Seetang et al. 2013; Zhou et al. 2020; Raddatz et al. 2022; Xu et al. 2023). Indeed, due to the colloidal nature of spherical AuNPs, they show characteristic surface resonance (SPR) absorption properties that typically peak at 520 nm (red color). This property makes AuNPs a useful colorimetric sensor widely used for the detection of several analytes by the naked eye (Jongjinakool et al. 2014), including nucleic acids. In fact, DNA-nanoprobes targeting specific nucleic acid sequences tend to aggregate in the presence of high salt concentrations unless they could bind to a specific target sequence by complementary hybridization that keeps their primary red color in solution. Otherwise, its aggregation leads to the formation of a new absorption band at longer wavelengths, eventually turning colorless.

In this study, we validated a proof-of-concept (POC) test for aMPV based on RT-LAMP using six specific primers combined with specific DNA-nanoprobes as a smart and simple readout approach. Moreover, this validated colorimetric system showed sensitivity and accuracy levels very close to qRT-PCR procedures for aMPV diagnosis. To our knowledge, this is the first RT-LAMP-based POC test for molecular detection of aMPV in situ.

## Materials and methods

### Sample compilation

A panel of 50 total nucleic acid samples extracted and purified from 15 upper respiratory tract swabs and 35 tracheal tissue samples, denoted as M1 to M50, collected from 34 chickens and 16 turkeys (Supplemental Table S1-A) were kindly provided by “Centro de Sanidad Avícola de Cataluña y Aragón” (CESAC) and analyzed. Out of 50 samples, 33 had been previously diagnosed as aMPV-positive by CESAC using a qRT-PCR screening test targeting the SH gene (Mescolini et al. 2021). Furthermore, 17 samples that tested negative for aMPV but positive for other respiratory viruses affecting poultry, such as IBV and ILTV, were also analyzed (Supplemental Table S1-A). Nucleic acid extractions were performed using the MagMAX FFPE DNA/RNA Ultra Kit in the automated robot KingFisher Flex (Thermo Fisher Scientific, Waltham, MA, USA) following the manufacturer’s instructions. Then, the products were aliquoted to prevent repetitive freezing and thawing cycles and stored at − 80 °C.

### Nucleic acid extraction from commercial vaccines

Live attenuated vaccines Nobilis® ND Clone 30 (Merck, Darmstadt, Germany); Hipraviar SHS, strain 1062 (HIPRA S.A., Amer, Spain); and Poulvac ILT, Salsbury strain (Zoetis Spain S.L., Spain), were kindly supplied by these companies and used as positive controls for ND virus (NDV), aMPV, and ILTV, respectively. Nobilis® ND Clone 30 vaccine, available in vials containing 1000 individual doses, was reconstituted following the manufacturer’s indications. Hipraviar SHS is available in vials with lyophilized suspension ranging from 10^2.4^ to 10^4.4^ TCID_50_ (50% tissue culture infection dose) and Poulvac ILT in vials with lyophilized suspension 146 ≥ 10^2.5^ EID_50_. Hipraviar SHS and Poulvac ILT vials were reconstituted in 1 mL of phosphate buffered saline (PBS), and total nucleic acids were purified using the GeneJET DNA/RNA Purification Kit (Thermo Fisher Scientific), following the manufacturer’s recommendations.

### RT-LAMP primers and oligonucleotide probe design

LAMP-primers and oligonucleotide probes for the aMPV-F gene were designed with PrimerExplorerV5 (https://primerexplorer.jp/e/) using default parameters and including loop primers (Supplemental Table S2). For their design, multiple alignment sequences of aMPV-A, aMPV-B, and aMPV-F genes (https://www.ncbi.nlm.nih.gov/nuccore) were performed using Clustal Omega (https://www.ebi.ac.uk/Tools/msa/clustalo/). Oligonucleotide LAMP primers used in this study (Supplemental Table S2) were synthesized by Macrogen Inc. (Seoul, South Korea).

Oligonucleotide probes used for AuNP functionalization were designed to target a conserved 17 nt-long sequence in the viral F gene (Supplemental Table S3). The designed probes were assessed for their secondary structure using the online RNAfold server (Vienna RNA Web Service; Gruber et al. 2008). Oligonucleotide probes with higher Gibbs free energy (Δ*G*_0_) values were chosen and synthetized. All oligonucleotide probes used in this study were synthesized by Sigma-Aldrich (Merck) and included a 5′-thiol modification.

### RT-LAMP and RT-qLAMP reaction settings

Purified viral RNA was amplified by one-step RT-LAMP (end-point RT-LAMP) or RT-qLAMP using WarmStart RTx Reverse Transcriptase and Bst 2.0 WarmStart® DNA Polymerase (New England Biolabs, Ipswich, MA, USA). Optimization of end-point RT-LAMP reaction was performed in 25 µL by mixing 12.5 µL of WarmStart MasterMix (WarmStart® Fluorescent LAMP/RT-LAMP Kit with uracil-DNA glycosylase (UDG), New England Biolabs), 1 µL of each primer set (optimization from 0.8 to 1.6 µM internal FIP/BIP primers, 0.1 to 0.4 µM outer F3/B3 primers, and 0.2 to 0.6 µM loop LF/LB primers), 20 U ribonuclease inhibitor (NZYTech, Lisbon, Portugal), and 5 µL of purified RNA. The reaction mix was prepared at room temperature to allow UDG activity, then incubated for 15 to 60 min at 60–65 °C for retrotranscription and Bst 2.0 amplification, followed by enzyme inactivation at 80 °C for 5 min in a Mastercycler® nexus GX2 thermocycler (Eppendorf, Hamburg, Germany). RT-LAMP reactions were visualized in 2% TAE-agarose gel electrophoresis.

For RT-qLAMP, 0.5 µL LAMP Fluorescent Dye (WarmStart® Fluorescent LAMP/RT-LAMP Kit, New England Biolabs) was included in the reaction mix. The fluorescence signal was monitored in a QuantStudio™ 5 Real-Time PCR System (Thermo Fisher Scientific), adding an end-point melt curve step to validate the specificity of amplification.

### Gold nanoparticle functionalization

AuNPs, 20 nm, supplied in citrate buffer solution (6.8 × 10^11^ nanoparticles/mL), were acquired from Nanovex Biotechnologies (Asturias, Spain). A solution of 6-mercapto-1-hexanol (MCH) and dithiotreitol (DTT)-reduced thiol probe-oligonucleotides (ratio 10:90, 2.5 µM final probe concentration) was incubated with 1 mL of AuNPs at room temperature for 16 h. The mixture was salt-aged up to 1.3 M NaCl, as previously described by Hurst et al. (2008) (Supplemental Fig. S1), with a few modifications. Briefly, the probe oligonucleotide and AuNP mixture were incubated with 0.2 M NaCl at room temperature for 2 h, then 0.01% Tween-20 was added, and finally subjected to vacuum centrifugation for 3–4 h at 40 °C to a final volume of 250 L. Afterwards, the DNA-nanoprobes were rinsed twice using 10 mM PBS (pH 7.5) at 10,000 rpm for 15 min at room temperature to remove unbound oligonucleotides. Finally, DNA-nanoprobes were resuspended in 10 mM PBS and conserved at 4 °C until further use.

### Detection by DNA-nanoprobes

To optimize colorimetric detection, 1.5 µL aMPV-F DNA-nanoprobes were incubated with 5 µL of end-point RT-LAMP products in a reaction solution buffered with 25 mM Tris at pH 7.5. The reaction buffer was supplemented with 2 M NaCl, 22 mM MgCl_2_, and 0.01% Tween-20. RNAse-free water was included as a negative control. Colorimetric detection was carried out on a thermal block at 37 °C for up to 90 min. The detection parameters were adjusted through absorbance readings at the 400–800 nm wavelength range taken at 15, 30, 45, 60, and 90 min post-incubation with DNA-nanoprobes using a NanoDrop 2000 Spectrophotometer (Thermo Fisher Scientific).

### Analytical sensitivity or limit of detection (LOD)

The aMPV-F gene was retrotranscribed and amplified in a one-step reaction by conventional RT-PCR using 10 µM of external RT-LAMP primers (F3 and B3), Affinity Script Multiple Temperature Reverse Transcriptase (Agilent Technologies, California, USA) for cDNA synthesis, and AmpliTaq DNA Polymerase (Thermo Fisher Scientific) for PCR amplification. The RT-PCR product was then cloned using the TOPO™ TA Cloning™ Kit for Sequencing (Thermo Fisher Scientific) and transformed into TOP10 Chemically Competent *Escherichia coli* (Thermo Fisher Scientific). Plasmid extraction was performed using the QIAamp DNA Mini Kit (Qiagen, Hamburg, Germany), and the aMPV-F gene was in vitro transcribed into RNA using T7 RNA Polymerase (Thermo Fisher Scientific). The RNA transcript was measured using the RNA Qubit (Thermo Fisher Scientific). To determine LOD, RT-qLAMP assays were performed on diluted RNA at 2.5 × 10^5^ copies. Then, fivefold dilutions were analyzed by RT-qLAMP along with DNA-nanoprobe detection in duplicate experiments.

## Results

### Optimization of RT-LAMP

**Fig. 1:**
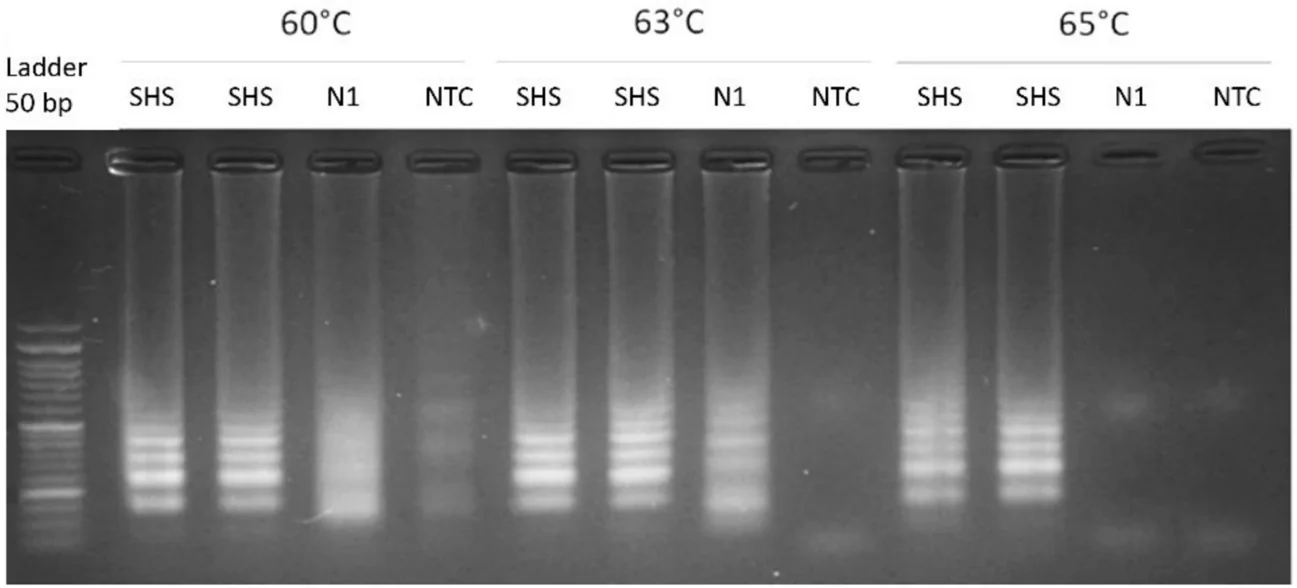
Representative DNA electrophoresis of end-point RT-LAMP products after 1-h reaction at temperatures of 60 °C, 63 °C, or 65 °C using the aMPV-F RT-LAMP. SHS, nucleic acids purified from aMPV vaccine in duplicate; N1, nucleic acids from ILTV vaccine (negative control); NTC, non-template control

To determine the optimal conditions for end-point RT-LAMP amplification, we tested a gradient of temperatures ranging from 60 to 65 °C using nucleic acids purified from the aMPV vaccine. Extracted nucleic acids from the ILTV vaccine were used as a negative control (Fig. 1).

**Fig. 2:**
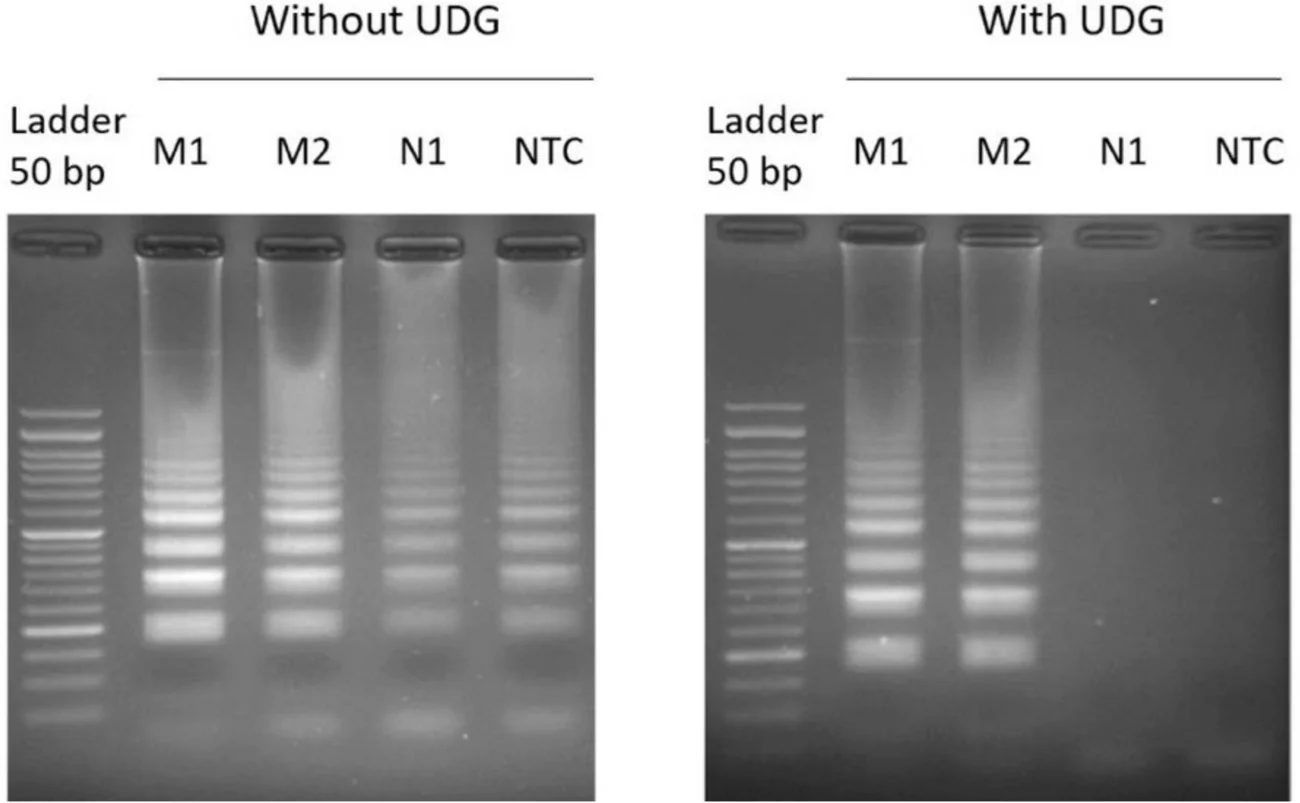
DNA electrophoresis of end-point RT-LAMP products at 65 °C for 30 min reaction using the aMPV-F RT-LAMP primer set without (left panel) or with UDG (right panel). M1 and M2, two positive aMPV samples; N1, ILTV vaccine (negative control); NTC, non-template control

As depicted in Fig. 1, specific amplification of aMPV RNA was observed at 65 °C. Of note, unspecific amplification for ILTV DNA was detected at both 60 °C and 63 °C. To fix this issue, further LAMP optimization was then assayed by including dUTP/UDG in the reaction mix. Since this enzyme effectively catalyzes the removal of any uracil nucleotides that may exist prior to the amplification reaction, we expected that any unspecific amplification due to carryover contamination would be prevented. In fact, negative controls showed no amplification under this condition (Fig. 2, right panel).

Subsequently, to set the optimal time conditions, the end-point RT-LAMP reaction, including UDG, was performed for 30 or 45 min at 65 °C, maintaining initial viral RNA concentrations. Since the fluorescence intensity of LAMP products peaked after 30 min (Supplemental Fig. S2), an optimal reaction time of 30 min for validation of RT-LAMP assays using aMPV samples was set. Besides, further optimization of primer concentration ranging from 0.025 to 2 µM each was also tested. According to our results, outer FIP/BIP primers at 1.6 µM, F3/B3 primers at 0.2 µM, and loop LF/LB primers at 0.4 µM exhibited optimal performance in LAMP reactions.

### Validation of RT-qLAMP assays for aMPV detection

**Fig. 3:**
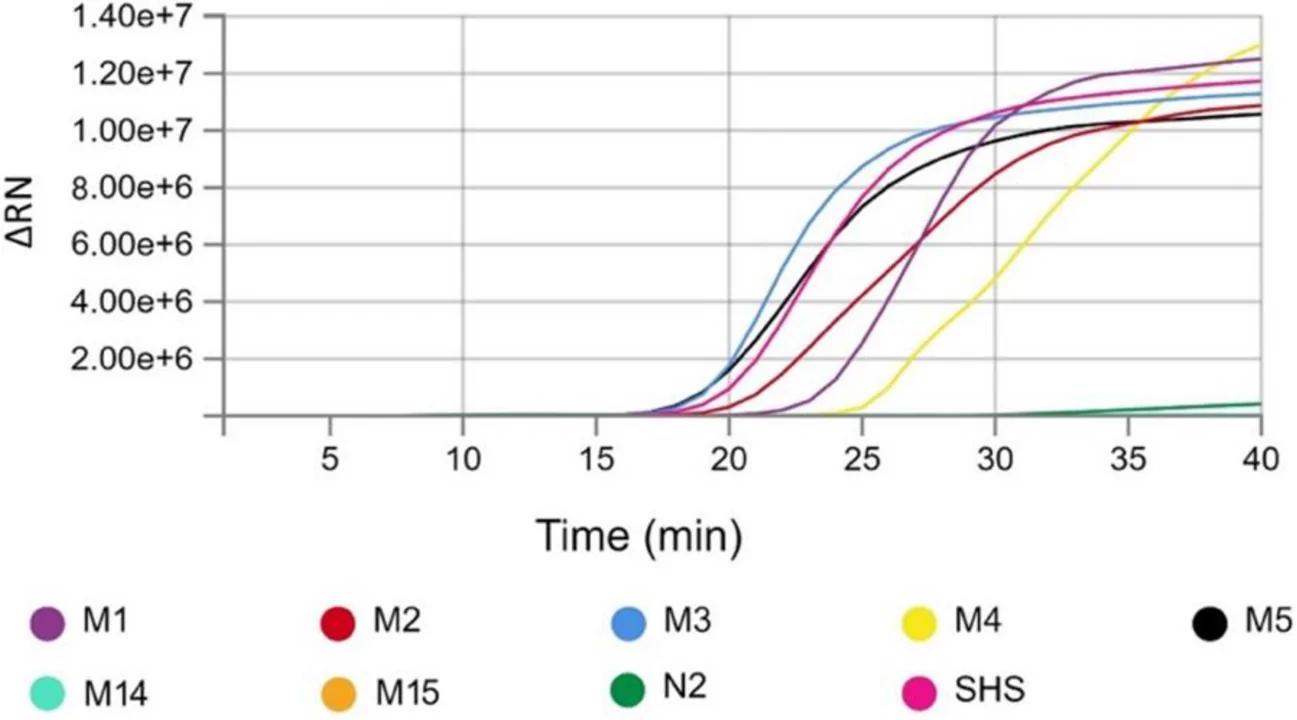
Representative RT-qLAMP amplification plot using aMPV-F LAMP primers and purified nucleic acids from several aMPV-positive samples (M1 to M5). To discard any potential cross-reactivity of the assay nucleic acids purified from IBV- and ILTV-positive samples, a live-attenuated vaccine for NDV was used (M14, M15, and N2 samples, respectively). Purified aMPV RNA from the SHS vaccine was used as a positive control

For sample validation and to verify the reaction specificity, RT-qLAMP was assayed with a melt curve step after DNA amplification. A panel of 50 RNA samples from both turkeys and chickens was analyzed (Supplemental Table S1-A). Our findings indicate that RT-qLAMP exhibited high sensitivity (87.9%, *n* = 29/33) and specificity levels (100%, *n* = 17/17) after 30 min of reaction (Fig. 3). Interestingly, we observed a 100% concordance between qRT-PCR and RT-qLAMP results when using turkey samples (*n* = 16/16). However, coincidence values for chicken samples were slightly lower than expected (88%, *n* = 30/34). Of note, there was full agreement between both tests in the chicken upper respiratory tract swabs (8/8) (Supplemental Table S1-B). Thus, discordant results were only observed when using tracheal tissues (8/12), probably due to repeated freezing/thawing of samples for RNA extraction.

### Validation of DNA-nanoprobe detection system coupled to RT-LAMP

**Fig. 4:**
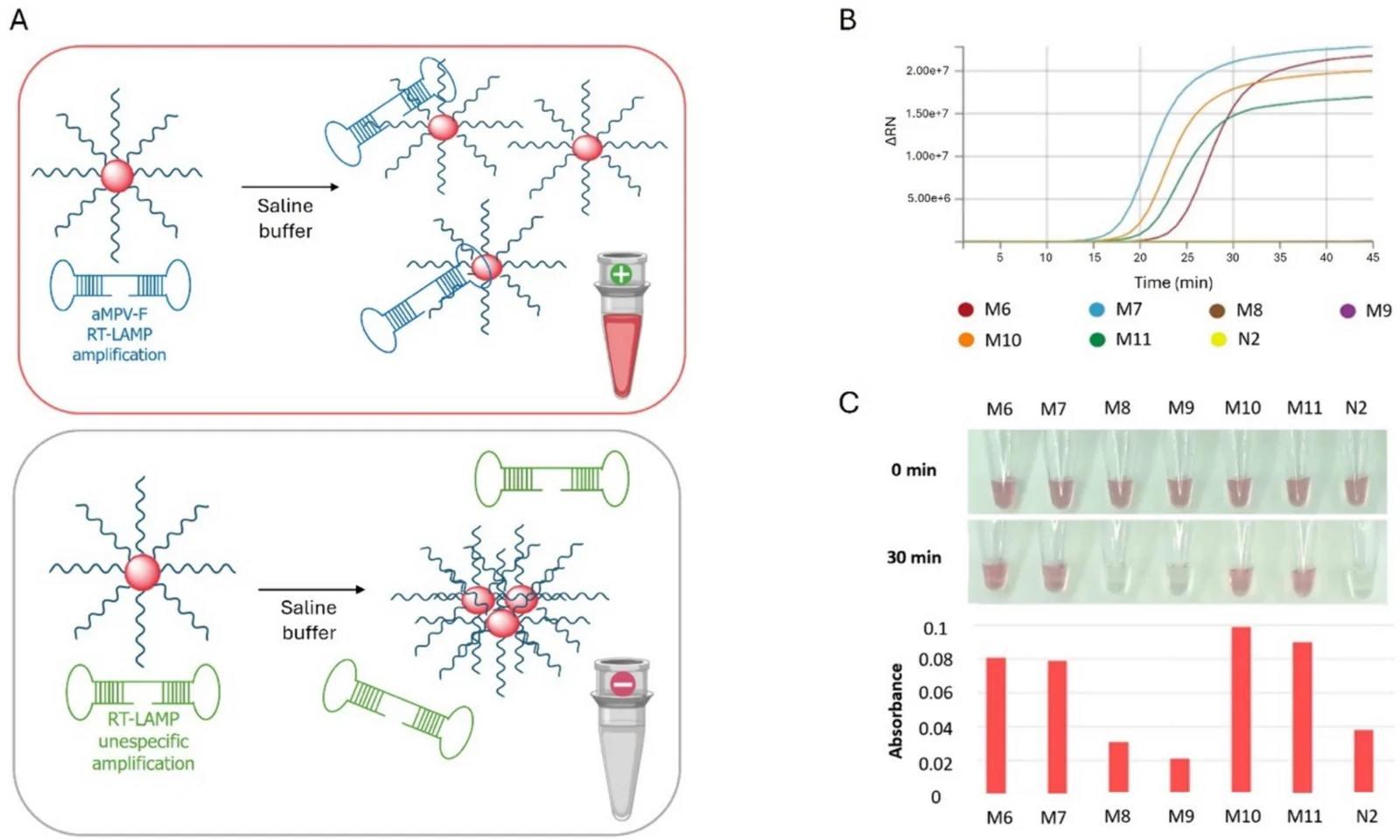
Validation of aMPV-F DNA-nanoprobes for visualization of RT-LAMP reactions targeting the aMPV-F gene. **A** Schematic representation of DNA-nanoprobe behavior depending on the presence of specific or unspecific RT-LAMP products. **B** RT-qLAMP amplification plot for 45 min of purified nucleic acids from representative aMPV samples (M6 to M11); purified RNA from NDV was used as a negative control (N2). **C** Digital pictures of reactions taken after 0 min or 30 min incubation at 37 °C of M6 to M11 RT-LAMP products with aMPV-F DNA-nanoprobes. Histograms represent their absorbance intensity at *λ* = 540 nm at 30 min of incubation at 37 °C

To assess the usefulness of the DNA-nanoprobes as a colorimetric tool for RT-LAMP readout, the end-point RT-LAMP products from the aMPV sample panel were incubated with the aMPV-F DNA-nanoprobes for up to 90 min at 37 °C (Fig. 4A). RT-qLAMP for aMPV-F was carried out in parallel (Fig. 4B) to validate RT-LAMP assays.

By visual examination of detection assays using specific aMPV-F DNA-nanoprobes (Fig. 4C), we found that the most distinct contrast between the positive and negative samples was established at a 5:1.5 (v/v) ratio of LAMP products to DNA-nanoprobes. During the incubation with DNA-nanoprobes, spectrophotometric values at UV-visible wavelengths were measured every 10 min. Remarkably, 30-min incubation was sufficient for identifying and distinguishing positive samples (Fig. 4C). The initial, red-colored DNA-nanoprobe suspension in the incubation buffer distinctively persisted throughout the reaction time in the presence of specific RT-LAMP products due to the target-dependent stabilization of nanoprobes. Conversely, reaction assays turned colorless in the absence of a specific target.

**Fig. 5:**
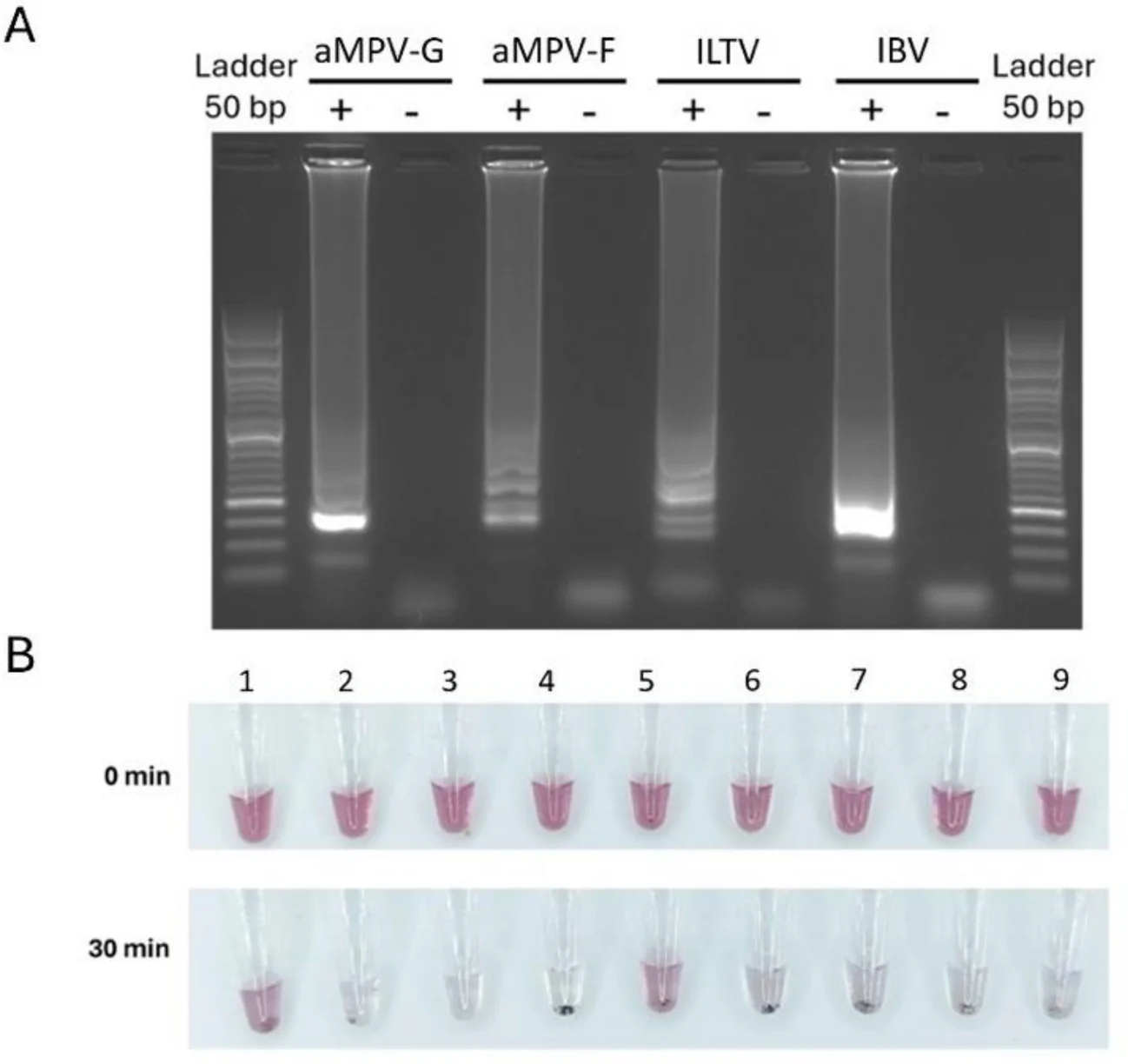
Representative results for the validation of the specificity of DNA-nanoprobes. **A** DNA electrophoresis of end-point RT-LAMP products at 65 °C for 30 min reaction using the indicated RT-LAMP primer sets for aMPV G-gene (lanes 2 and 3), aMP-F gene (lanes 4 and 5), ILTV-TK gene (lanes 6 and 7), or IBV-5′UTR (lanes 8 and 9) using their corresponding purified viral genome, or non-template controls ( −). **B** Binding results after incubation at 37 °C for 0 min or 30 min of respective RT-LAMP products with different DNA-nanoprobes. Tube 1, aMPV-F DNA-nanoprobe with an aMPV-F RT-LAMP product. Tube 2, competition assay of aMPV-F DNA-nanoprobe with an aMPV-F RT-LAMP product in the presence of 100 nM of a free aMPV-F oligonucleotide probe. Tube 3, non-functionalized AuNPs with an aMPV-F RT-LAMP product. Tube 4, aMPV-F DNA-nanoprobes with an aMPV-F RT-LAMP product of a negative sample. Tube 5, ILTV-TK DNA-nanoprobes with an ILTV-TK RT-LAMP product. Tube 6, ILTV-TK DNA-nanoprobes with an aMPV-F RT-LAMP product. Tube 7, aMPV-F DNA-nanoprobes with an ILTV-TK RT-LAMP product. Tube 8, aMPV-F DNA-nanoprobes with an IBV-5′UTR RT-LAMP product. Tube 9, aMPV-F DNA-nanoprobes with an aMPV-G RT-LAMP product

To confirm the aMPV-F DNA-nanoprobes as a reliable read-out system for detection of aMPV by RT-LAMP, we tested their specificity (Fig. 5). For this purpose, aMPV-F nanoprobes were incubated with either specific or unspecific RT-LAMP products (Fig. 5A), or, conversely, aMPV-F RT-LAMP products were tested using off-target DNA nanoprobes targeting the ILTV-TK (thymidine kinase) gene or the IBV-5′UTR. As shown in Fig. 5B, aMPV-F DNA-nanoprobes did not detect any RT-LAMP products but the aMPV-F amplification. Moreover, spike-in assays with 100 nM of free aMPV-F oligonucleotide probe abolished specific detection of the aMPV-F RT-LAMP product by aMPV-F DNA-nanoprobes, presumably through competitive binding.

The percentages of sensitivity, specificity, and accuracy of the RT-LAMP coupled to the DNA-nanoprobes system for molecular detection of aMPV were calculated using a conventional confusion matrix (Table 1). Of note, only 4 out of 33 positive aMPV samples (M9, M39, M41, and M48 samples) were determined to be false negatives.

**Table 1:** Performance parameters obtained from the confusion matrix for aMPV-F RT-LAMP and DNA-nanoprobe detection system and confidence interval of 95% (95% CI)

Statistic	Value	95% CI
Sensitivity	87.88%	71.80 to 96.60%
Specificity	100.00%	80.49 to 100.00%
Accuracy	92.00%	80.77 to 97.78%

**Fig. 6:**
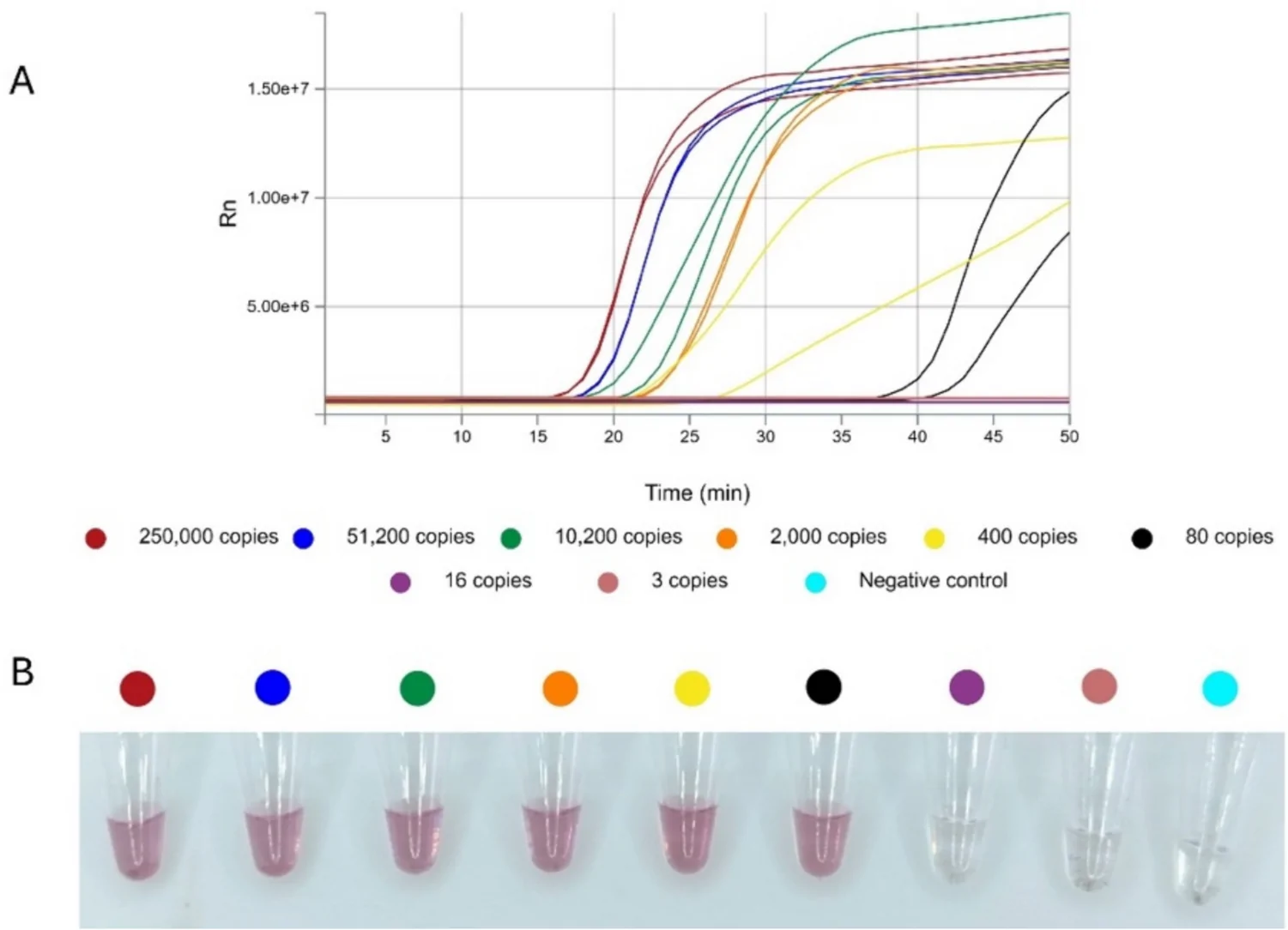
Results for the LOD determination assay using aMPV-F DNA-nanoprobes. **A** aMPV-F RT-qLAMP amplification plot after 50 min of incubation using fivefold dilutions of transcripted RNA from the TOPO-aMPV-F gene construct. **B** Binding results after 0 min or 30 min incubation at 37 °C of respective RT-qLAMP products with aMPV-F DNA-nanoprobes

Finally, we calculated the analytical sensitivity or LOD of the system by combining RT-LAMP and specific aMPV-F DNA-nanoprobes. As shown in Fig. 6, using serial dilutions of in vitro transcribed RNA, its LOD was 80 copies/reaction (16 copies/μL).

Therefore, we propose a definitive protocol for specific and reliable molecular detection of aMPV by RT-LAMP combined with DNA-nanoprobes. The amplification reaction should contain 12.5 µL of WarmStart MasterMix with dUTP/UDG, 20 U of RNAse inhibitor, 1.6 µM of primers FIP/BIP, 0.2 µM for F3/B3, 0.4 µM for LF/BF, and 5 µL of purified total nucleic acids for 25 µL reactions. Reactions are carried out for 30 min at 65 °C, with a final inactivation at 80 °C for 5 min. Results are then visualized by the incubation of 5 µL of RT-LAMP products and 1.5 µL of DNA-nanoprobes (50 µM) in the incubation buffer (2 M NaCl, 22 mM MgCl_2_, and 0.01% Tween-20) for 30 min at 37 °C.

## Discussion

In this study, we have developed and validated a method based on RT-LAMP amplification combined with specific DNA-nanoprobes for the detection of aMPV using field samples. According to our results, the combination of RT-LAMP procedures, using up to three pairs of specific oligonucleotides and DNA-nanoprobes targeting discrete regions of the viral genome, attains sensitivity and specificity levels on par with qRT-PCR methods, which have been the gold standard in molecular detection this far. Therefore, a system combining RT-LAMP and specific DNA-nanoprobes could be easily implemented as a cost-effective and reliable system that could be widely implemented for in situ screening, not only of aMPV but also of other viruses. This system has already been developed for the detection of human and animal viruses (Kampeera et al. 2021; Ye et al. 2022), but it is not yet well implemented for livestock viruses at the farm level.

The high sensitivity levels of LAMP are one of its main virtues as a potential alternative for molecular detection as POC devices, notably in developing countries or rural areas without basic health care infrastructures. However, some drawbacks of this technique have been widely reported, including potential cross-contamination (Tomita et al. 2008; Morris et al. 2015; Bao et al. 2020). To prevent this issue, current RT-LAMP kits have been improved by adding an UDG enzyme (Lai et al. 2022), still designed as a close-tube reaction without increasing amplification time. This enzyme promotes the degradation of uracil-nucleotides in pre-amplified products but without any effect on the original template. In our study, the usefulness of this strategy has been successfully attained since the number of false positives was virtually reduced to 0% using field samples.

While previous studies have developed diagnostic methods for other avian viruses (Padzil et al. 2021), this is the first study describing an RT-LAMP test for the molecular detection of aMPV. The clinical diagnosis of aMPV in field conditions is challenging due to co-infections and the similarity of clinical signs with other respiratory infections. Currently, RT-PCR and qRT-PCR tests have become the established gold standard methods in reference laboratories for diagnosing active infections. Most of the RT-PCR epidemiological studies on aMPV are based on M, N, and G genes (Ferreira et al. 2009; Kariithi et al. 2022; Wang et al. 2022). In this study, we targeted its F gene based on its high conservation degree in the reference strains of aMPV subtypes A and B. A recent study supports that these subtypes, especially aMPV-B, are the most prevalent not only in Spain but also in many European countries (Mescolini et al. 2021).

A drawback of the LAMP-based strategy as a reliable POC detection system lies in the visualization procedure required to identify and differentiate positive from negative samples. Various methods have been described so far that do not need equipment, enabling diagnosis by the naked eye. One of the first approaches measures the reaction turbidity at end-point LAMP amplification. In fact, as the reaction progresses, magnesium pyrophosphate is generated and precipitates when positive samples undergo amplification (Mori et al. 2001). Nevertheless, to attain a discernible precipitate, this approach depends on both high amplification efficiency and a significant product yield, and samples with low viral loads might be classified as false negatives. Other LAMP systems use colorimetric indicators, such as hydroxynaphthol blue, a metal indicator that turns from violet to blue as the free Mg^2+^ concentration decreases when positive samples are successfully amplified. Despite that, this method has very low sensitivity levels, approximately 10–100 ng of RNA (Hongjaisee et al. 2021), and low specificity values, around 80%, compared to gold standard qRT-PCR (Prakash et al. 2023). Phenol- or cresol-red are also pH indicators used as a read-out for LAMP-based diagnosis (Huang et al. 2020). These pH indicators can detect the release of protons during DNA synthesis, resulting in a color shift from pink to yellow in positive samples. While Raddatz et al. (2022) improved this system for SARS-CoV-2 detection, the slight pH changes registered during LAMP amplification may be one of the major limitations for its use as a POC detection system. This limitation can pose significant challenges, especially when dealing with unprocessed samples. In general, LAMP-based diagnostic technologies show decreased specificity levels, which can result in a higher rate of false positive outcomes.

Fluorescent-based LAMP detection systems have also been proposed as an alternative to qPCR-based ones. For instance, it has been recently described as a multiplexed system for the detection of foot-and-mouth disease, vesicular stomatitis, and bluetongue viruses in a single reaction tube (Fan et al. 2022). This fluorescent-based multiplex LAMP shows a detection limit of 526 copies for recombinant plasmids, and it is not compatible enough for POC implementation as it requires well-trained personnel as well as sophisticated and expensive equipment. Of note, Padzil et al. (2021) describe a fluorescent-based LAMP system for avian influenza virus detection showing impressive sensitivity levels (LOD > 10 copies/reaction). Despite this, these systems are not as economically viable as POC tests due to the expensive fluorescent-conjugated primers required. Our system shows a high analytical sensitivity or LOD, as it can detect ≥ 80 RNA copies/reaction (16 copies/μL). Based on the time required for amplification of the gene F RNA, it is conceivable that field samples would carry a viral load of ≥ 80 copies per swap or gram of tissue. Therefore, sampling asymptomatic birds, which might harbor lower viral loads or viral RNA degradation due to freeze–thaw processes, could contribute to the false positives found in our study.

In terms of the utility of AuNP in diagnostics, recent studies detail detection systems, primarily for human pathogens, that integrate RT-LAMP and lateral flow biosensors with AuNPs (Zhu et al. 2020; Chen et al. 2021; Akalin and Yazgan-Karatas 2023). However, the combination of non-functionalized AuNPs and biosensors based on fluorescently labeled LAMP primers (Zhu et al. 2020; Chen et al. 2021) may result in a colorimetric detection of decreased specificity.

Unlike these approaches, the colorimetric system validated in the current study for aMPV detection is highly specific as it couples precise LAMP-based reactions to 100% homologous DNA-nanoprobes as a read-out. The main drawback of the present methodology relies on the requirement of a two-step open-tube approach, which would potentially increase the risk of cross-contamination. However, our system relies on colorimetric detection that is specific to the viral RNA sequence, effectively mitigating the occurrence of false positive results during the LAMP reaction. Additionally, it prevents the detection of nonspecific amplifications or artifacts arising from primer dimer formation (Padzil et al. 2021). This way, it overcomes the limitations of previous one-step LAMP visualization methods, such as hydroxynaphthol blue, turbidimetry, or pH-based systems, that suffer from low sensitivity, especially when working with biological samples in the field. Compared with fluorescent methods, DNA-nanoprobes do not require any special equipment, greatly reducing costs. Thus, the proposed system can be used as a useful colorimetric method for molecular detection of aMPV RNA by the naked eye in just 60 min: 30 min of RT-LAMP plus 30 min of incubation with DNA-nanoprobes. Therefore, we suggest its usefulness as a POC detection system for early, rapid, simple, and cost-effective specific screening for aMPV in situ*.* However, a highly desirable approach would be the development of a multiplexed LAMP strategy for the detection of viral respiratory co-infections and/or those that exhibit similar clinical signs to aMPV infection, irrespective of whether the read-out device is used.

In summary, in this work, we have developed a potential plasmonic biosensor that is easy to perform in resource-limited settings based on the combination of RT-LAMP and colorimetric detection using DNA-nanoprobes. This system has been shown to be reliable, accurate, and faster when compared to qRT-PCR setups. Furthermore, this detection platform exhibits considerable potential for development as a POC diagnostic system through the utilization of a partial lysis buffer to facilitate accessibility to viral RNA. Given its easy implementation for the detection of other pathogens, our approach has the potential to enhance accessibility to cost-effective diagnostic devices for on-site mass screening, addressing a broad spectrum of clinically or veterinary-relevant RNA-based viruses. This system, fully compatible with on-farm diagnostics, can be used for large-scale screening by simply implementing a plate reader incorporating a 540 nm filter, thus allowing the automated reading of results.

## Supplementary Information

Below is the link to the electronic supplementary material.

Supplementary file1 (PDF 230 KB)
