# Supplementary material for: An affordable detection system based on RT-LAMP and DNA-nanoprobes for avian metapneumovirus
Source: Appl Microbiol Biotechnol. 2024 Jul 10;108:414. doi: 10.1007/s00253-024-13243-x (PMC11236856; doi:10.1007/s00253-024-13243-x)
Supplement: Supplementary file 1 [file 253_2024_13243_MOESM1_ESM.pdf]

Supplemental data

Applied Microbiology and Biotechnology

An Affordable Detection System based on RT-LAMP and DNA-Nanoprobes for Avian Metapneumovirus

Pablo Cea-Callejo[1,2]†, Sonia Arca-Lafuente[1]†, Esperanza Gomez-Lucia[2,3], Ana Doménech[,3], Mar Biarnés[4], Angela Blanco[4], Laura Benítez*[5]‡; Ricardo Madrid*[1,2,5]‡.

[1]BioAssays SL. Parque Científico de Madrid, Campus de Cantoblanco, Madrid, Spain

[2]Research Group of "Animal Viruses" of Complutense University of Madrid

[3]Deparment of Animal Health, Veterinary Faculty, Complutense University of Madrid (UCM), Spain

[4]Centro de Sanidad Avícola de Cataluña y Aragón (CESAC)

[5]Department of Genetics, Physiology, and Microbiology, School of Biology, Complutense University of Madrid (UCM), Spain

†These authors contributed equally to this work and share first authorship

‡ These authors contributed equally to this work and share last authorship

* Correspondence: Ricardo Madrid (rimadrid@ucm.es), Laura Benítez (lbenitez@ucm.es)

Supplemental Table S1-A: Summary of the comparative analysis for detection of aMPV in different type of samples. Samples tested as negative but positive for Infectious Bronchitis virus (IBV) or Infectious Laryngothacheitis Virus (ILTV) are in parentheses.

Sample code	Specie	Type of sample	RT-qPCR	RT-LAMP/ DNA-nanoprobes
M1	Turkey	Tracheal tissue	+	+
M2	Turkey	Pharyngeal swab	+	+
M3	Turkey	Tracheal tissue	+	+
M4	Turkey	Pharyngeal swab	+	+
M5	Chicken	Pharyngeal swab	+	+
M6	Chicken	Pharyngeal swab	+	+
M7	Chicken	Tracheal tissue	+	+
M8	Chicken	Tracheal tissue	- (IBV)	-
M9	Chicken	Tracheal tissue	+	-
M10	Chicken	Tracheal tissue	+	+
M11	Chicken	Tracheal tissue	+	+
M12	Turkey	Tracheal tissue	+	+
M13	Turkey	Tracheal tissue	+	+
M14	Chicken	Pharyngeal swab	- (IBV)	-
M15	Chicken	Pharyngeal swab	- (ILT)	-
M16	Chicken	Pharyngeal swab	+	+
M17	Chicken	Tracheal tissue	- (ILT)	-
M18	Chicken	Pharyngeal swab	+	+
M19	Chicken	Pharyngeal swab	+	+
M20	Turkey	Tracheal tissue	+	+
M21	Turkey	Tracheal tissue	+	+
M22	Turkey	Pharyngeal swab	+	+
M23	Turkey	Tracheal tissue	+	+

M24	Turkey	Tracheal tissue	+	+
M25	Chicken	Tracheal tissue	-	-
M26	Chicken	Tracheal tissue	+	+
M27	Chicken	Tracheal tissue	+	+
M28	Turkey	Tracheal tissue	+	+
M29	Turkey	Tracheal tissue	- (IBV)	-
M30	Turkey	Tracheal tissue	- (IBV)	-
M31	Turkey	Tracheal tissue	+	+
M32	Turkey	Tracheal tissue	- (IBV)	-
M33	Chicken	Pharyngeal swab	- (IBV)	-
M34	Chicken	Tracheal tissue	- (ILT)	-
M35	Chicken	Pharyngeal swab	+	+
M36	Chicken	Pharyngeal swab	+	+
M37	Chicken	Tracheal tissue	- (IBV)	-
M38	Chicken	Tracheal tissue	- (IBV)	-
M39	Chicken	Tracheal tissue	+	-
M40	Chicken	Tracheal tissue	+	+
M41	Chicken	Tracheal tissue	+	-
M42	Chicken	Pharyngeal swab	- (IBV)	-
M43	Chicken	Tracheal tissue	- (ILT)	-
M44	Chicken	Tracheal tissue	- (IBV)	-
M45	Chicken	Tracheal tissue	+	+
M46	Chicken	Tracheal tissue	+	+
M47	Chicken	Tracheal tissue	- (ILT)	-
M48	Chicken	Tracheal tissue	+	-
M49	Chicken	Pharyngeal swab	+	+
M50	Chicken	Tracheal tissue	- (ILT)	-

Supplemental Table S1-B: Summary of RNA sample panel characteristics based on qRT-PCR screening using the aMPV-SH gene. Discrepancies obtained by RT-LAMP coupled to the DNA nanoprobe detection system (gen F) are indicated in parentheses.

	Swabs (+)	Swabs (-)	Tissues (+)	Tissues (-)	**TOTAL**
RNA PANEL	15		35		
Chickens	8	4	12 (8)	10	**34 (30)**
Turkeys	3	0	10	3	**16**
Subtotal	**11**	**4**	**22 (18)**	**13**	
**TOTAL**	**33 (29) positives+17 negatives**				**50**

Supplemental Table S2: Sequences of the primer sets used for aMPV viral RNA amplification by RT-LAMP.

**Target virus**	**Primer set**	**Primer name**	**Sequence (5'-3')**
**aMPV**	aMPV-genF	F3-aMPV	GTAATGATGGTCCTAGCCTTA
		B3-aMPV	GCTTTCACCTCCCCTTCT
		FIP-aMPV	CGATTCTCCTTTGTAATCTGATCGGAATTATCACTAACTCAGAATGCCT
		BIP-aMPV	GAAGAGGTTTGTGTTGGGTGCTTTAGCTAAAGCTACGCCG
		LoopF-aMPV	GAAACAGTTCTAAGCTCCTGCA
		LoopB-aMPV	CACCACAGCTGCTGTAACAG

Supplemental Table S3: DNA oligonucleotide-probe sequences designed for aMPV detection.

Target virus	Oligonucleotide probe name	Sequence (5’-3’)
**aMPV**	aMPV-gene-F	TTTTTTTTTTAGGAGCTTAGGCTAGCTACAACTGTTTCT

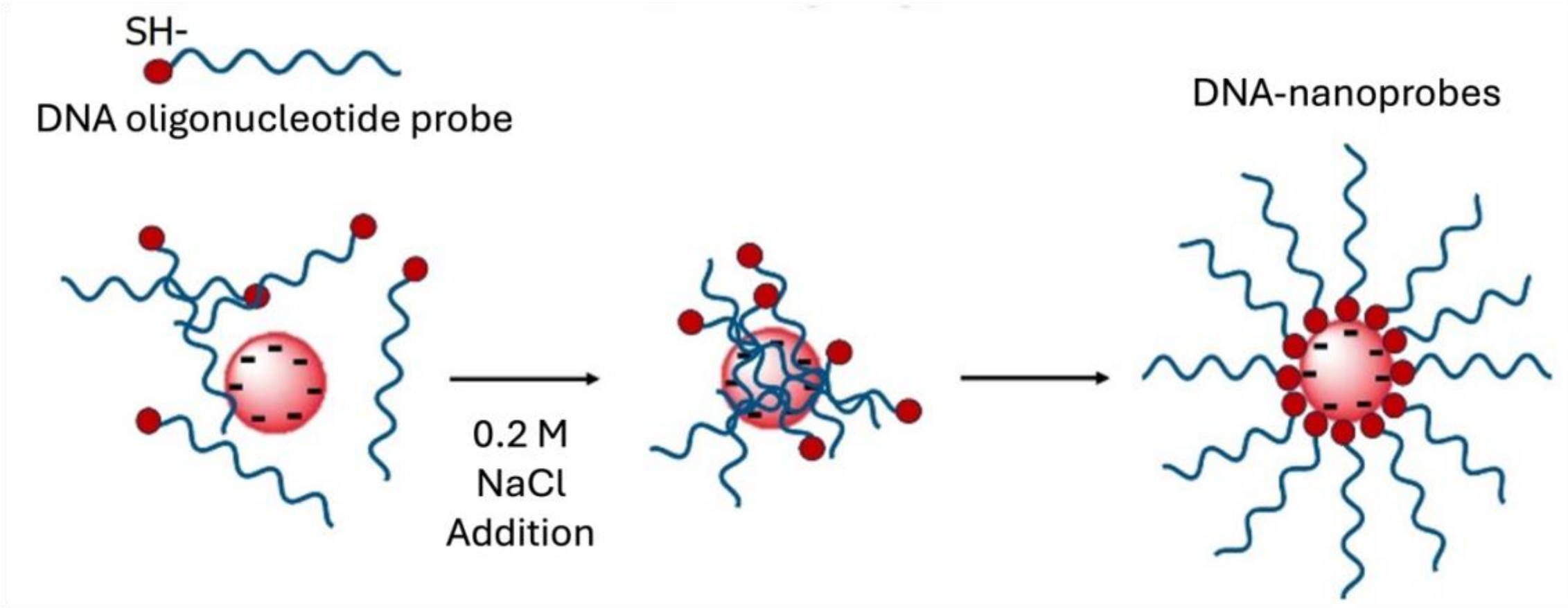

Supplemental Fig. S1. Salt aging functionalization of gold nanoparticles (AuNPs) with DNA oligonucleotide probe.

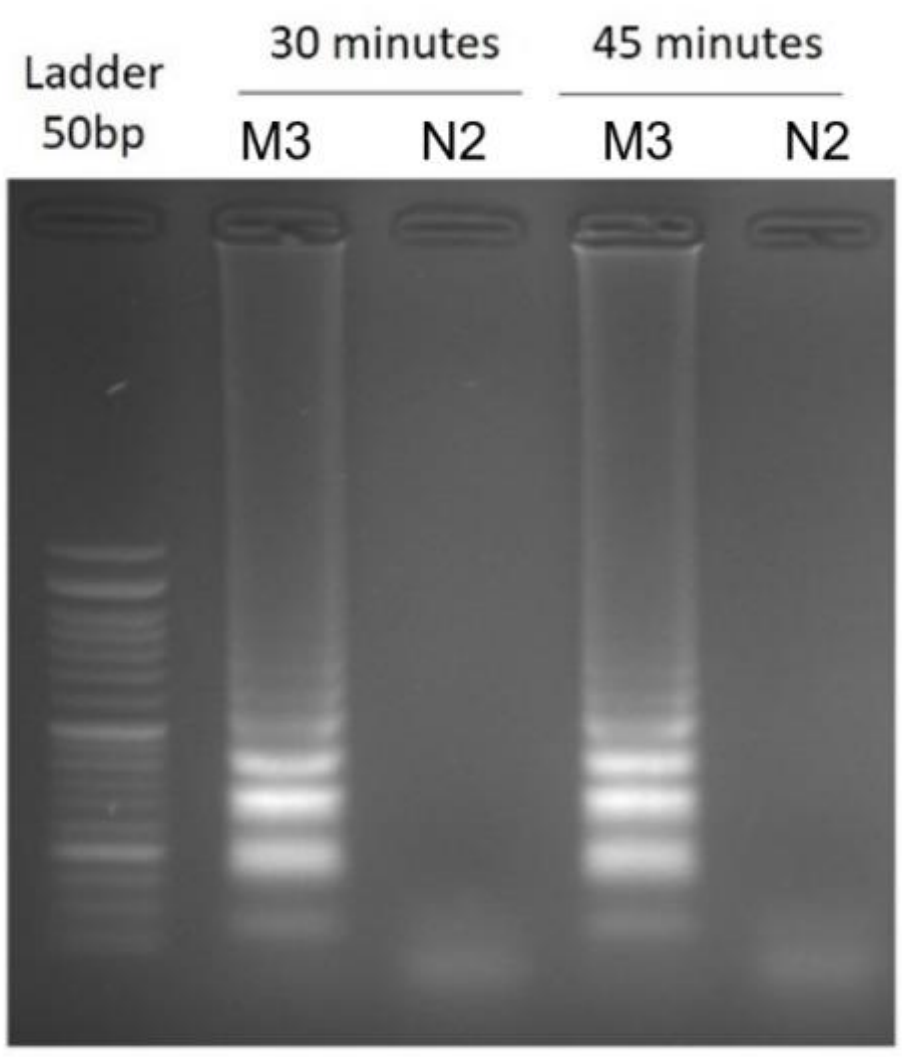

Supplemental Fig. S2. End-point RT-LAMP reaction with the primer set targeting the aMPV sequence gene testing two different reaction times (30 and 45 min) at 65°C. M3 is an isolated RNA from positive aMPV sample and N2 from ILTV RNA used as negative control.
